# Unvaccinated periodontal patients with a history of COVID-19: clinical findings in a Dental School setting

**DOI:** 10.1590/0103-6440202305284

**Published:** 2023-12-22

**Authors:** Vanessa B. Roza, Sabrina C. Brasil, Luiza F. Mello, Carina M. Silva-Boghossian

**Affiliations:** 1 Postgraduate Program in Dentistry, Universidade do Grande Rio, Professor José de Souza Herdy, 1160, Jardim Vinte e Cinco de Agosto, Duque de Caxias, RJ, Brazil; 2 School of Dentistry, Dental Clinic Department, Universidade Federal do Rio de Janeiro Rio de Janeiro, R. Prof. Rodolpho Paulo Rocco, 325, Cidade Universitária, Rio de Janeiro, RJ, Brazil.

**Keywords:** COVID-19, periodontal diseases, periodontal pocket, periodontal attachment loss

## Abstract

This study analyzed the periodontal clinical data of individuals with a history of COVID-19 treated in a dental school during the pandemic in 2021 before vaccination. Methods: This analysis included individuals older than 18 years with no history of systemic disorders other than systemic arterial hypertension. Individuals who had COVID-19 were classified according to the World Health Organization as asymptomatic, with mild, moderate, severe, or critical symptoms. Results: A total of 95 individuals were evaluated, which included 24 with a history of COVID-19. Seventeen percent had been asymptomatic, 21% had mild, 25% moderate, 21% severe, and 17% critical symptoms, including intubation. Individuals with no history of COVID-19 presented significantly lower measurements of probing depth (p=0.003; Mann-Whitney test) and clinical attachment level (p=0.002) compared to individuals with a history of COVID-19. A significant negative association was found between bleeding on probing and the severity of characteristics of COVID-19 (rho= -0.233; p=0.023). Conversely, positive associations between the values of probing depth (rho= 0.292; p=0.004) and mean clinical attachment level (rho= 0.300; p=0.003) and the characteristics of COVID-19 were found. Conclusions: The periodontal data shows that patients who had COVID-19 before vaccination may present a worse periodontal status when compared to patients in the same clinical setting with no history of COVID-19. However, a more extensive study should confirm it with more participants.

## Resumo

Este estudo analisou os dados clínicos periodontais de indivíduos com histórico de COVID-19 tratados em uma escola de odontologia durante a pandemia em 2021, antes da vacinação.

**Métodos:**

Essa análise incluiu indivíduos maiores de 18 anos sem histórico de distúrbios sistêmicos, exceto hipertensão arterial sistêmica. Os indivíduos que tiveram COVID-19 foram classificados de acordo com a Organização Mundial da Saúde como assintomáticos, com sintomas leves, moderados, graves ou críticos.

**Resultados:**

95 indivíduos foram avaliados, incluindo 24 com histórico de COVID-19. 17% eram assintomáticos, 21% tinham sintomas leves, 25% moderados, 21% graves e 17% críticos, incluindo intubação. Os indivíduos sem histórico de COVID-19 apresentaram medidas significativamente mais baixas de profundidade de sondagem (p=0,003; teste de Mann-Whitney) e nível de fixação clínica (p=0,002) em comparação com indivíduos com histórico de COVID-19. Foi encontrada uma associação negativa significativa entre o sangramento à sondagem e a gravidade das características da COVID-19 (rho= -0,233; p=0,023). Por outro lado, foram encontradas associações positivas entre os valores de profundidade de sondagem (rho = 0,292; p = 0,004) e o nível médio de apego clínico (rho = 0,300; p = 0,003) e as características da COVID-19.

**Conclusão:**

Os dados periodontais mostraram que os pacientes que tiveram COVID-19 antes da vacinação podem apresentar um pior estado periodontal quando comparados a pacientes no mesmo ambiente clínico sem histórico de COVID-19. No entanto, um estudo mais extenso deve ser realizado para confirmar tal achado com maior número de participantes.

## Introduction

The pandemic of Coronavirus infectious disease 2019 (COVID-19) was declared by the World Health Organization in March 2020. It is caused by the severe acute respiratory syndrome coronavirus 2 (SARS-CoV-2). ^1^ This disease is especially severe when the patient presents with comorbidities, which include diabetes, obesity, and advanced age. ^1^, ^2^ Growing evidence has also associated periodontitis with the severity of the clinical state of COVID-19 patients. ^3^ ^),(^ ^4^ ^),(^ ^5^ ^),(^ ^6^ ^),(^ ^7^

SARS-CoV-2 can be found in many sites in the oral cavity, such as mucosal cells and tissues, saliva, supra and subgingival biofilms, and gingival crevicular fluid. ^8^ ^),(^ ^9^ ^),(^ ^10^ As the virus has an airborne transmission, dental care had to undergo several adaptations to avoid its dissemination. ^11^ For biosafety reasons, Dental Schools were closed for patients for several months. By mid-2021, almost all Dental Schools were re-opening due to the increase in the rate of the vaccinated population. Some private Dental Schools were re-structured to attend patients by the end of 2020, even before vaccination had started in Brazil. ^12^ This period of interruption in dental care or periodontal maintenance, especially for periodontal patients, may have impacted negatively the onset and progression of the disease.^13^

In Brazil, the vaccination against SARS-CoV-2 started slowly only by the end of January 2021. ^12^ As the data collection was in March 2021, the current investigation was able to collect data from individuals who had not had the opportunity to get vaccinated at the time. Moreover, patients were returning to dental care over a year of service interruption. Therefore, this study aimed to analyze periodontal clinical data of individuals with a history of COVID-19 treated in a dental school during the pandemic in 2021 before vaccination.

## Material and methods

The population of the study was recruited from individuals who were seeking treatment at the periodontics clinic at the Dental School of the Universidade do Grande Rio from March to May 2021. The study protocol was explained to patients and written informed consent was obtained from all participants. The study followed the Helsinki Declaration of Human Studies and received approval from the Ethics Committee of the Universidade do Grande Rio (# 4.226.744).

Included individuals were adults (>18 years old) from both sexes. Exclusion criteria included individuals with current pregnancy or lactation, autoimmune diseases, hepatitis, kidney disease, and human immunodeficiency virus infection.

### Clinical evaluation

After dental and medical history recording, individuals were subjected to a complete periodontal examination. Clinical examination was performed at six sites per tooth at all teeth, excluding third molars. One trained and calibrated examiner (V.B.R.) performed the examinations. The intra-class correlation coefficients for clinical attachment level and probing depth were 0.96 and 0.95, respectively. The clinical examination included dichotomous measures, the presence or absence, of dental calculus, supragingival biofilm, suppuration, and bleeding on probing (BOP), as well as measurement in mm of probing depth (PD) and clinical attachment level (CAL), using a North Carolina periodontal probe (Hu-Friedy, Chicago, IL, USA). PD measured the distance between the gingival margin and the most apically probable portion, while CAL measured the distance from the enamel junction to the most apically probable portion. Periodontal diagnoses followed the guidelines of the current periodontal classification ^14^. Periodontal health was identified when the patient had PD in all sites ≤ 3 mm, assuming no pseudo periodontal pockets, and BOP < 10% of the sites. Gingivitis was defined when patients had a BOP of ≥ 10%. Both periodontal health and gingivitis patients could present CAL, although without the presence of BOP in the same site. Periodontitis patients had at least two non-adjacent interdental sites with CAL at ≥2mm with BOP. Interdental CAL is detectable at ≥2 non-adjacent teeth, or buccal CAL ≥3 mm with pocketing >3 mm detectable at ≥2 teeth. Besides, the observed CAL could not be ascribed to non-periodontal causes as listed in Tonetti et al.^15^. All individuals received periodontal treatment after evaluation.

### History of COVID-19

Individuals with a history of COVID-19 were classified according to the World Health Organization as asymptomatic (i.e., positive laboratory test but no clinical symptoms); mild symptoms (i.e., nonspecific symptoms, such as fever or chills, cough, loss of taste or smell, diarrhea, abdominal pain, muscle or body aches, headache and/or tiredness); moderate symptoms (i.e., persistent cough and fever, prostration, loss of appetite, presence of pneumonia); severe symptoms (i.e., Severe Acute Respiratory Syndrome, involving dyspnea/ respiratory distress or persistent pressure in the chest or oxygen saturation below 95% or pale, gray, or blue-colored skin, lips, or nail beds, depending on skin tone); and critical symptoms (i.e., need for respiratory support and admissions to intensive care units) (https://www.gov.br/saude/pt-br/coronavirus/sintomas).

### Data analysis

Current data were analyzed using a statistical package (IBM SPSS Statistics for Windows, Version 22.0. Armonk, NY: IBM Corp.). The Kolmogorov-Smirnov normality test demonstrated that clinical variables did not a normal distribution. Individuals’ demographic characteristics were analyzed by group, including mean age, distribution of sex, percentage of smokers, and frequency of gingivitis and periodontitis. The frequency of sites with BOP, suppuration, dental calculus, and supragingival biofilm was obtained, as well as the median (interquartile range) of the PD and the CAL. The PD and CAL data were analyzed for their extent according to the current periodontal classification ^14^. The values of PD were categorized as shallow (0-3 mm), moderate (4-6 mm), and deep (>6 mm), while CAL was categorized as incipient (0-2 mm), moderate (3-4 mm) and severe (≥ 5 mm). Clinical data were calculated by each individual and then across the group. For analysis purposes, individuals with mild or moderate symptoms of COVID-19 were grouped in one group, and individuals with severe or critical symptoms were grouped in another. Therefore, part of the analysis considered three groups of individuals with a history of COVID-19 instead of five groups. Significant differences were tested by Chi-square, Kruskal-Wallis, and Mann-Whitney tests. Spearman’s correlation coefficient was used to analyze associations between the categories of the classification of COVID-19 and periodontal clinical parameters. The significance level was established at 5%.

## Results

A total of 95 individuals were evaluated, which included 24 with a history of COVID-19 and 71 with no history of that disease. There was no significant difference between those groups of participants in terms of mean age, distribution of sex, percentage of smokers, and percentage of individuals with systemic arterial hypertension (Table 1). The frequency of individuals with gingivitis or periodontitis was similar. Clinical parameters, including the percentage of BOP and suppuration as well as median values of the full-mouth PD and CAL, differed significantly between participants when grouped according to the history of COVID-19 (p < 0.05; Mann-Whitney test), although no significant difference was found for the remaining parameters. 

**Table 1:** Participants’ demographic and periodontal characteristics according to a history of COVID-19.

Variable	History of COVID-19 (n = 24)	No history of COVID-19 (n = 71)	P value *
Mean age (± SD)	41.3 (9.4)	45.8 (11.1)	>0.05 ^†^
% Women	41.7	57.7	>0.05
% Smokers	62.5	43.7	>0.05
*Systemic disease (%)*			
None	79.2	76.1	>0.05
Systemic arterial hypertension	20.8	23.9	
% Gingivitis	54.2	47.9	>0.05
% Periodontitis	45.8	52.1	
*Periodontal parameters (Median; IQR)*			
% Dental calculus	25.5 (31.4)	18.1 (41.1)	>0.05
% Dental biofilm	39.8 (24,9)	29.2 (19.1)	>0.05
% BOP	18.1 (9.1)	22.7 (14.2)	0.023
% Suppuration	1.4 (11.9)	0 (1.9)	0.04
PD (mm)	3.7 (1.9)	2.5 (1.7)	0.003
CAL (mm)	4.3 (2.6)	2.8 (2.3)	0.002
% Shallow PD	94.3 (42.3)	72.2 (53.2)	>0.05
% Moderate PD	5.7 (40.2)	25.1 (41.3)	>0.05
% Deep PD	0 (7.9)	0.4 (10.2)	>0.05
% Incipient CAL	38.6 (47.1)	34.9 (43.6)	>0.05
% Moderate CAL	38.5 (16.2)	39.1 (14.5)	>0.05
% Severe CAL	12.9 (41.9)	23.7 (47.9)	>0.05

*Chi-square test; ^†^ Mann-Whitney test; ^‡^ Other systemic diseases: hypothyroidism (n=3), anemia (n=1), hypothyroidism (n=4), bronchitis (n=1); SD: standard-deviation; IQR: interquartile range; BOP: bleeding on probing; PD: Probing depth, CAL: Clinical attachment level

**Figure 1:**
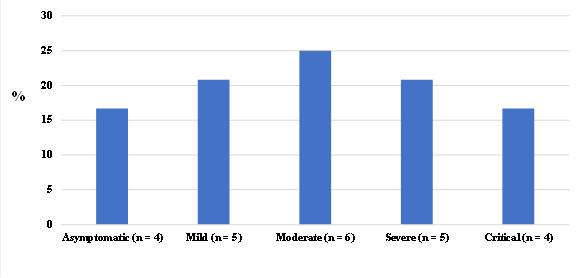
Distribution of individuals according to the characteristics of COVID-19. Asymptomatic (i.e., positive laboratory test but no clinical symptoms); mild symptoms (i.e., nonspecific symptoms, such as fever or chills, cough, loss of taste or smell, diarrhea, abdominal pain, muscle or body aches, headache and/or tiredness); moderate symptoms (i.e., persistent cough and fever, prostration, loss of appetite, presence of pneumonia); severe symptoms (i.e., Severe Acute Respiratory Syndrome, involving dyspnea/ respiratory distress or persistent pressure in the chest or oxygen saturation below 95% or pale, gray, or blue-colored skin, lips, or nail beds, depending on skin tone); and critical symptoms (i.e., need for respiratory support and admissions to intensive care units).

Figure 1 presents the distribution of individuals with a history of COVID-19 according to their symptoms. Seventeen percent were asymptomatic, 21% had mild symptoms, 25% had moderate symptoms, 21% had severe symptoms, and 17% had critical symptoms, including intubation.

Significant associations between clinical periodontal parameters and characteristics of COVID-19 are presented in Table 2. A negative significant association was found between BOP and the severity of characteristics of COVID-19 (*rho* = -0.233; p = 0.023). Conversely, positive associations between values of PD (*rho* = 0.292; p = 0.004) and CAL (*rho* = 0.300; p = 0.003) and the characteristics of COVID-19 were found.

**Table 2:** Correlation analysis between characteristics of COVID-19 and periodontal parameters.

Periodontal parameters	Characteristics of COVID-19 *	
	*rho* ^†^	P value
%BOP	-0.233	0.023
PD	0.292	0.004
CAL	0.300	0.003

* Categories of COVD-19: 1 - Asymptomatic; 2 - Mild symptoms; 3 - Moderate symptoms; 4 - Severe symptoms; 5 - Critical symptoms. ^†^ *rho*: Spearman’s correlation coefficient. BOP: bleeding on probing; PD: probing depth; CAL: clinical attachment level. Only significant correlations are presented.

## Discussion

This is a secondary analysis of a study in which the relationship between periodontal status and hypertension was investigated in the periodontics clinic at a dental school. The current analysis intended to report some clinical findings that caught our attention. In special, we demonstrated that the evaluated individuals with a history of COVID-19 had the worst periodontal condition concerning individuals without a history of COVID-19. As it is a secondary analysis, we performed a power analysis using the current values of the PD and CAL considering differences between the two groups, with or without a history of COVID-19. In brief, using specific software (WinPepi, http://www.brixtonhealth.com, Jerusalem, Israel), our findings had a power of 96.8% and 83.6% when a difference of 1 mm in PD or CAL was detected between the two groups, respectively. Even reaching the appropriate study power, the current investigation does include a low number of COVID-19 cases. However, we would like to stress that the investigation occurred in the first semester of 2021, a period of the pinnacle of cases of COVID-19 when despite the existence of the vaccine against Sarsc-cov-2, it was not available to everyone. In that context, the imposed isolation and physical distancing, the access of patients to Dental Schools was allowed only after a series of restrictions and on a much lower scale than we used to have on a daily basis. All those setbacks explain the low number of individuals included in our investigation, although one should keep in mind that clinical studies back then were almost impossible to perform.

As Brazil was one of the countries with relatively high dissemination of the SARS-Cov-2, it is no surprise that we had a significant number of patients at the beginning of 2021 with a history of COVID-19. The patients were enrolled before they had taken the vaccine against SARS-CoV-2. In Brazil, vaccination started in January 2021 and the elderly individuals were prioritized, starting with individuals 90 or more years of age.^12^ By May 2021, which was the final month when the clinical part of the study was carried on, only 19.4% of the adult population had taken at least one dose of the vaccine where the referred Dental School is located. ^16^ Also in May 2021, the mean death due to COVID-19 was reaching almost 300 individuals per day in the state of Rio de Janeiro. ^17^

There is a crescent number of studies that shows that periodontitis increases the chance of severe COVID-19.^4^, ^6^, ^7^ On the other hand, patients who had been treated for periodontitis and had their supportive periodontal therapy suspended due to the pandemic, presented a worsening in their periodontal condition. ^13^ The current studied individuals were patients who had no treatment for periodontitis for at least one and a half years, which can be considered non-treated periodontitis. The collected data demonstrated that the parameters PD and CAL were positively associated with the severity of symptoms of COVID-19, while BOP was negatively associated. One hypothesis is that individuals who had the more severe forms of the disease received a high load of antibiotics and anti-inflammatory/ corticosteroid drugs, which can reduce clinical periodontal signs of inflammation, such as BOP. In xxx, it is not new that antibiotics are usually overprescribed without microbiological proof of infection.^18^ A further increase in their administration was evident because of COVID-19 not only in hospitalized individuals but also in self-medication.^19^ On the other hand, PD and CAL are not directly affected by systemic antimicrobials when used without mechanical periodontal treatment. ^20^

In conclusion, the periodontal data shows that patients who had COVID-19 before vaccination may present a worst of periodontal status when compared to patients in the same clinical setting with no history of COVID-19. However, a more extensive study should confirm it with more participants.
